# Why Biopsychosocial Determinants Matter: Is Age Just a Number?

**DOI:** 10.1093/geroni/igaa057.2201

**Published:** 2020-12-16

**Authors:** Andrew Steptoe

## Abstract

Aging is a physiological and dynamic process enduring time, which is influenced by various underlying mechanisms occurring within the biological, psychological and social spheres. We investigated biopsychosocial determinants of subsequent health and mortality in the English Longitudinal Study of Ageing (ELSA) (Steptoe). Cognitive impairment and dementia, particularly Alzheimer’s disease (AD), represent significant challenges to individuals, families and healthcare. We found an indication of socioeconomic differentials influencing the mediating biological and psychological pathways in relation to subsequent cognitive health, which was ascertained with a latent g factor across various cognitive domains in the Harmonised Cognitive Assessment Protocol in ELSA (Cadar). We also identified an interplay between socioeconomic markers and genetic factors influencing the time of dementia and Alzheimer’s disease (AD) diagnosis in individuals from the English Longitudinal Study of Ageing, particularly in those with a polygenetic predisposition to AD (Ajnakina). Within the same cohort, we found that participants who transitioned into a single household due to divorce or bereavement had a higher risk of mortality (Abell). The adverse health outcomes associated with loneliness are well documented, but less is known in terms of hospitalization and accessing health care. In the Healthy Ageing in Scotland (HAGIS), we found an increased hospitalization for older individuals reporting higher loneliness (Douglas); and various loneliness patterns in relation to age, gender, marital status and socioeconomic status in participants from first wave of the Northern Ireland Cohort for the Longitudinal Study of Ageing (NICOLA) (Neville). Our findings highlight the imperative need for policy interventions and tailored strategies.

